# (6S*)-6-[(1S*,2R*)-1,2-Di­hydroxy­pent­yl]-4-meth­oxy-5,6-di­hydro-2*H*-pyran-2-one

**DOI:** 10.1107/S1600536813027025

**Published:** 2013-10-19

**Authors:** Domenic J. Valenti, Atta M. Arif, Gary A. Strobel, James K. Harper

**Affiliations:** a Department of Chemistry, University of Central Florida, 4104 Libra Drive, Orlando, FL 32816, USA; b University of Utah, Department of Chemistry, 315 S. 1400 E. Rm. 2020, Salt Lake City, UT 84112, USA; c Department of Plant Sciences and Plant Pathology, Montana State University, 206 Plant Bioscience Building, Bozeman, MT 59717, USA

## Abstract

The title compound, C_11_H_18_O_5_, was isolated from a liquid culture of *Pestalotiopsis sp*. In the mol­ecule, the pyran-2-one ring assumes a half-chair conformation. The two terminal C atoms of the pentyl group were refined as disordered over two sets of sites, with refined occupancies of 0.881 (10) and 0.119 (10). In the crystal, mol­ecules are linked *via* O—H⋯O hydrogen bonds forming a three-dimensional network.

## Related literature

For the first isolation of the title compound, see: McGahren *et al.* (1973 ▶). For the natural and unnatural stereospecific synthesis, see: Kirihata *et al.* (1990 ▶, 1992*a* ▶,*b* ▶); Masaki *et al.* (1994 ▶). For closely related products from other fungi, see: Kimura *et al.* (1986 ▶); Kirihata *et al.* (1996 ▶); Lee *et al.* (1995 ▶); Davies-Coleman & Rivett (1989 ▶). For biological activity, see: Venkatasubbaiah & Van Dyke (1991 ▶). For crystal structures of related compounds, see: Yoshino & Nowacki (1972 ▶); Engel & Nowacki (1972*a* ▶,*b* ▶).
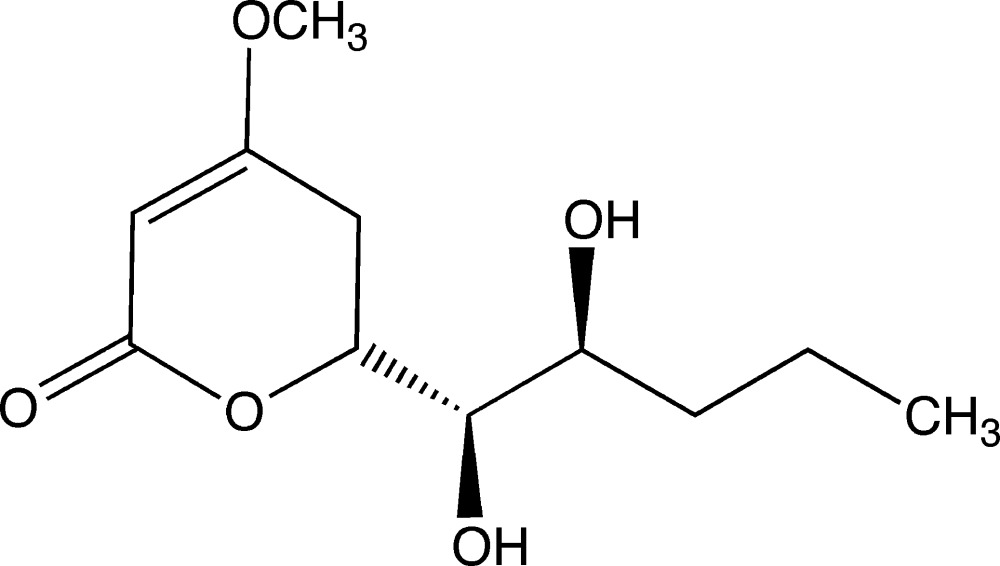


## Experimental

### 

#### Crystal data

C_11_H_18_O_5_
*M* *_r_* = 230.25
Orthorhombic, 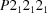

*a* = 5.0375 (3) Å
*b* = 11.4515 (13) Å
*c* = 20.802 (2) Å
*V* = 1200.02 (19) Å^3^
*Z* = 4
Mo *K*α radiation
μ = 0.10 mm^−1^
*T* = 200 K
0.28 × 0.18 × 0.08 mm

#### Data collection

Nonius KappaCCD diffractometer
Absorption correction: multi-scan (*DENZO-SMN*; Otwinowski & Minor, 1997 ▶) *T*_min_ = 0.973, *T*_max_ = 0.992
2711 measured reflections
1616 independent reflections
1153 reflections with *I* > 2σ(*I*)
*R*_int_ = 0.043

#### Refinement

*R*[*F* ^2^ > 2σ(*F* ^2^)] = 0.052
*wR*(*F* ^2^) = 0.121
*S* = 1.06
1616 reflections
188 parameters
H atoms treated by a mixture of independent and constrained refinement
Δρ_max_ = 0.16 e Å^−3^
Δρ_min_ = −0.18 e Å^−3^

### 

Data collection: *COLLECT* (Nonius, 1998 ▶); cell refinement: *DENZO-SMN* (Otwinowski & Minor, 1997 ▶); data reduction: *DENZO-SMN*; program(s) used to solve structure: *SIR97* (Altomare *et al.*, 1999 ▶); program(s) used to refine structure: *SHELXL97* (Sheldrick, 2008 ▶); molecular graphics: *WinGX* (Farrugia, 2012 ▶), *ORTEP-3 for Windows* (Farrugia, 2012 ▶) and *PLATON* (Spek, 2009 ▶); software used to prepare material for publication: *SHELXL97*.

## Supplementary Material

Crystal structure: contains datablock(s) I, global. DOI: 10.1107/S1600536813027025/lh5653sup1.cif

Structure factors: contains datablock(s) I. DOI: 10.1107/S1600536813027025/lh5653Isup2.hkl

Supplementary material file. DOI: 10.1107/S1600536813027025/lh5653Isup3.cml

Additional supplementary materials:  crystallographic information; 3D view; checkCIF report

## Figures and Tables

**Table 1:** Hydrogen-bond geometry (Å, °)

*D*—H⋯*A*	*D*—H	H⋯*A*	*D*⋯*A*	*D*—H⋯*A*
O1′—H1′*O*⋯O2^i^	0.85 (3)	1.93 (3)	2.778 (3)	177 (3)
O2′—H2′*O*⋯O2′^ii^	0.80 (4)	2.05 (4)	2.8178 (18)	163 (4)

Symmetry codes: (i) 
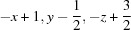
; (ii) 
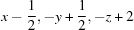
.

**Fig. 1.**
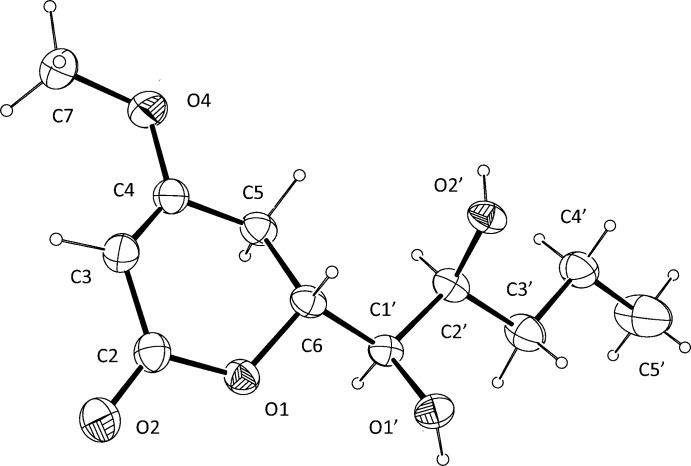
Molecular structure of the title compound. Displacement ellipsoids are shown at the 50% probability level on non-hydrogen atoms. The disorder is not shown.

**Fig. 2.**
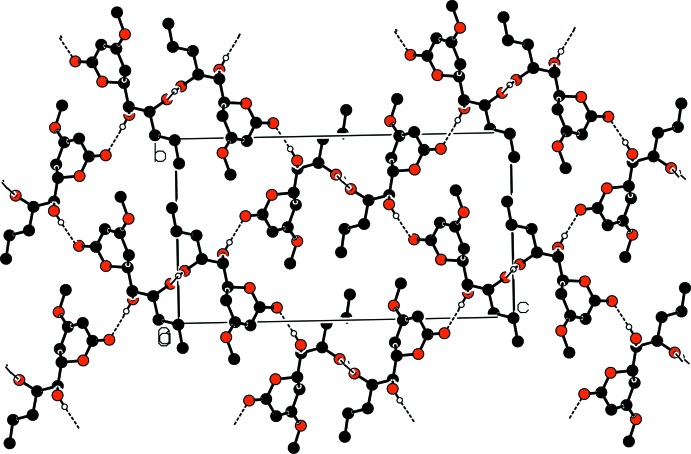
A portion of the crystal structure viewed along the a axis. The dashed lines indicate O—H···O hydrogen bonds. The disorder is not shown.

## supplementary crystallographic information

### 1. Comment

The title compound was first isolated from an unidentified *Penicillium sp*. (McGahren *et al.*, 1973). Multiple routes have been reported for the total synthesis (Kirihata *et al.*, 1990; Kirihata *et al.*, 1992*a*; Masaki *et al.*, 1994) including syntheses creating unnatral stereoisomers (Kirihata *et al.*, 1992*b*). Closely related products have been reported from other fungi (Kirihata *et al.*, 1996; Lee *et al.*, 1995). The 6-substituted 5,6-dihydropyran-2-one moiety present in the title compound is also found in natural products from several species of plants and fungi (Davies-Coleman & Rivett, 1989). Although many structures with this moiety exhibit bioactivity, it appears that the title compound displays none of the reported activities. Notably, the gibberellin synergistic activity of the very closely related compound pestalotin is not found (Kimura *et al.*, 1986; Venkatasubbaiah & Van Dyke, 1991). In our lab we observed moderate antifungal activity. Crystal structures for the related natural products, kavain, dihydrokavain and methysticin have been reported (Yoshino & Nowacki, 1972; Engel & Nowacki, 1972*a*; Engel & Nowacki, 1972*b*). In the title compound, the atoms of the pyran-2-one assume a half-chair conformation. The conformations of the methoxy and dihydroxypentyl groups are shown in Fig. 1. Carbons 4' and 5' are disordered over two sites with occupancies of 0.881 (10):0.119 (10). In the crystal, molecules are linked via O—H···O hydrogen bonds forming a three-dimensional network (see Table 1 and Fig. 2).

### 2. Experimental

The title compound was obtained by liquid-liquid extraction (CH_2_Cl_2_/H_2_O) of a culture of an endophytic *Pestalotiopsis sp*. The CH_2_Cl_2_ fraction was evaporated under reduced pressure then purified by chromatography on a silica column with CHCl_3_/CH_3_OH (8/2) as eluent. After pooling common fractions, a crystal was grown by slow evaporation of a MeOH solution.

### 3. Refinement

The molecule exhibits orientational disorder at atoms C4 and C5. Hydrogen atoms were located and refined isotropically except those on C4 and C5 which were placed in calculated positions of C—H = 0.98 and 0.99Å and assigned isotropic displacement parameters of U_iso_(H) = 1.2U_eq_(C) or 1.5U_eq_(C_methyl_), and their coordinates were allowed to ride on their respective carbons using *SHELXL97* (Sheldrick, 2008). In the absence of anomalous dispersion effects the Friedel pairs were merged. The absolute configuration is not known.

### Crystal data

**Table:**

C_11_H_18_O_5_	*F*(000) = 496
*M**_r_* = 230.25	*D*_x_ = 1.274 Mg m^−^^3^
Orthorhombic, *P*2_1_2_1_2_1_	Mo *K*α radiation, λ = 0.71073 Å
Hall symbol: P 2ac 2ab	Cell parameters from 17230 reflections
*a* = 5.0375 (3) Å	θ = 1.0–27.5°
*b* = 11.4515 (13) Å	µ = 0.10 mm^−^^1^
*c* = 20.802 (2) Å	*T* = 200 K
*V* = 1200.02 (19) Å^3^	Plate, colorless
*Z* = 4	0.28 × 0.18 × 0.08 mm

### Data collection

**Table:**

Nonius KappaCCD diffractometer	1616 independent reflections
Radiation source: fine-focus sealed tube	1153 reflections with *I* > 2σ(*I*)
Graphite monochromator	*R*_int_ = 0.043
φ plus ω scans	θ_max_ = 27.5°, θ_min_ = 3.4°
Absorption correction: multi-scan (*DENZO-SMN*; Otwinowski & Minor, 1997)	*h* = −6→6
*T*_min_ = 0.973, *T*_max_ = 0.992	*k* = −14→14
2711 measured reflections	*l* = −26→26

### Refinement

**Table:**

Refinement on *F*^2^	Secondary atom site location: difference Fourier map
Least-squares matrix: full	Hydrogen site location: inferred from neighbouring sites
*R*[*F*^2^ > 2σ(*F*^2^)] = 0.052	H atoms treated by a mixture of independent and constrained refinement
*wR*(*F*^2^) = 0.121	*w* = 1/[σ^2^(*F*_o_^2^) + (0.0544*P*)^2^ + 0.1236*P*] where *P* = (*F*_o_^2^ + 2*F*_c_^2^)/3
*S* = 1.06	(Δ/σ)_max_ < 0.001
1616 reflections	Δρ_max_ = 0.16 e Å^−^^3^
188 parameters	Δρ_min_ = −0.18 e Å^−^^3^
0 restraints	Extinction correction: *SHELXL97* (Sheldrick, 2008), Fc^*^=kFc[1+0.001xFc^2^λ^3^/sin(2θ)]^-1/4^
Primary atom site location: structure-invariant direct methods	Extinction coefficient: 0.033 (6)

### Special details

**Table:**

Experimental. The program DENZO-SMN (Otwinowski & Minor, 1997) uses a scaling algorithm that effectively corrects for absorption effects. High redundancy data were used in the scaling program thus the 'multi-scan' code word was used. No transmission coefficients are available from the program (only scale factors for each frame). The scale factors in the experimental table are calculated from the 'size' command in the SHELXL97<i/> input file.
Geometry. All e.s.d.'s (except the e.s.d. in the dihedral angle between two l.s. planes) are estimated using the full covariance matrix. The cell e.s.d.'s are taken into account individually in the estimation of e.s.d.'s in distances, angles and torsion angles; correlations between e.s.d.'s in cell parameters are only used when they are defined by crystal symmetry. An approximate (isotropic) treatment of cell e.s.d.'s is used for estimating e.s.d.'s involving l.s. planes.
Refinement. Refinement of *F*^2^ against ALL reflections. The weighted *R*-factor *wR* and goodness of fit *S* are based on *F*^2^, conventional *R*-factors *R* are based on *F*, with *F* set to zero for negative *F*^2^. The threshold expression of *F*^2^ >σ(*F*^2^) is used only for calculating *R*-factors(gt) *etc* and is not relevant to the choice of reflections for refinement. *R* factors based on *F*^2^ are statistically about twice as large as those based on *F*, and *R*-factors based on ALL data will be even larger.

### Fractional atomic coordinates and isotropic or equivalent isotropic displacement parameters (Å^2^)

**Table:**

	*x*	*y*	*z*	*U*_iso_*/*U*_eq_	Occ. (<1)
O1	0.4082 (4)	0.31378 (17)	0.78556 (9)	0.0429 (5)	
O2	0.5372 (5)	0.4250 (2)	0.70604 (12)	0.0678 (7)	
O4	−0.0742 (6)	0.5734 (2)	0.85714 (11)	0.0685 (8)	
O1'	0.5361 (4)	0.1294 (2)	0.86362 (10)	0.0451 (6)	
H1'O	0.516 (7)	0.066 (3)	0.8434 (15)	0.050 (10)*	
O2'	0.2491 (5)	0.21272 (19)	0.97762 (9)	0.0443 (5)	
H2'O	0.125 (8)	0.238 (3)	0.9970 (17)	0.051 (10)*	
C2	0.4146 (7)	0.4194 (3)	0.75666 (15)	0.0481 (8)	
C3	0.2702 (8)	0.5156 (3)	0.78511 (15)	0.0541 (8)	
H3	0.302 (8)	0.590 (3)	0.7637 (17)	0.070 (11)*	
C4	0.0890 (7)	0.4946 (3)	0.83017 (13)	0.0520 (8)	
C5	0.0460 (6)	0.3748 (3)	0.85552 (15)	0.0454 (7)	
H5A	−0.004 (7)	0.381 (2)	0.9013 (15)	0.048 (8)*	
H5B	−0.094 (8)	0.335 (3)	0.8314 (15)	0.056 (9)*	
C6	0.3007 (6)	0.3053 (3)	0.85045 (13)	0.0395 (7)	
H6	0.433 (6)	0.340 (2)	0.8789 (12)	0.028 (6)*	
C1'	0.2742 (5)	0.1766 (3)	0.86336 (14)	0.0376 (6)	
H1'	0.168 (5)	0.146 (2)	0.8299 (12)	0.026 (7)*	
C2'	0.1275 (6)	0.1490 (3)	0.92562 (14)	0.0421 (7)	
H2'	−0.057 (6)	0.174 (3)	0.9211 (14)	0.044 (8)*	
C3'A	0.1175 (9)	0.0209 (3)	0.94034 (14)	0.0598 (9)	0.881 (10)
H3'A	0.3000	−0.0079	0.9481	0.072*	0.881 (10)
H3'B	0.0454	−0.0213	0.9027	0.072*	0.881 (10)
C4'A	−0.0549 (13)	−0.0058 (5)	0.99940 (18)	0.0624 (14)	0.881 (10)
H4'A	0.0249	0.0315	1.0377	0.075*	0.881 (10)
H4'B	−0.2335	0.0283	0.9930	0.075*	0.881 (10)
C5'A	−0.0814 (18)	−0.1325 (4)	1.0111 (3)	0.118 (3)	0.881 (10)
H5'A	−0.1905	−0.1454	1.0495	0.177*	0.881 (10)
H5'B	0.0949	−0.1666	1.0178	0.177*	0.881 (10)
H5'C	−0.1660	−0.1695	0.9739	0.177*	0.881 (10)
C3'B	0.1175 (9)	0.0209 (3)	0.94034 (14)	0.0598 (9)	0.119 (10)
H3'C	0.2915	−0.0094	0.9258	0.072*	0.119 (10)
H3'D	−0.0147	−0.0109	0.9098	0.072*	0.119 (10)
C4'B	0.064 (7)	−0.043 (3)	1.0025 (13)	0.036 (7)*	0.119 (10)
H4'C	0.1133	0.0035	1.0409	0.043*	0.119 (10)
H4'D	0.1484	−0.1207	1.0041	0.043*	0.119 (10)
C5'B	−0.244 (5)	−0.049 (2)	0.9920 (11)	0.038 (8)*	0.119 (10)
H5'D	−0.3267	−0.0887	1.0287	0.057*	0.119 (10)
H5'E	−0.2819	−0.0932	0.9526	0.057*	0.119 (10)
H5'F	−0.3161	0.0299	0.9881	0.057*	0.119 (10)
C7	−0.0519 (12)	0.6930 (3)	0.83475 (18)	0.0924 (17)	
H7A	−0.1803	0.7420	0.8577	0.139*	
H7B	−0.0884	0.6960	0.7885	0.139*	
H7C	0.1282	0.7218	0.8430	0.139*	

### Atomic displacement parameters (Å^2^)

**Table:**

	*U*^11^	*U*^22^	*U*^33^	*U*^12^	*U*^13^	*U*^23^
O1	0.0439 (11)	0.0449 (12)	0.0398 (10)	−0.0023 (9)	0.0045 (9)	0.0010 (9)
O2	0.0802 (18)	0.0588 (15)	0.0645 (14)	0.0010 (13)	0.0250 (14)	0.0148 (12)
O4	0.0831 (18)	0.0673 (15)	0.0549 (13)	0.0342 (14)	−0.0025 (13)	−0.0027 (11)
O1'	0.0373 (11)	0.0457 (13)	0.0525 (12)	0.0014 (9)	0.0025 (9)	−0.0052 (11)
O2'	0.0365 (11)	0.0575 (13)	0.0388 (10)	−0.0012 (11)	−0.0009 (9)	−0.0102 (9)
C2	0.0505 (18)	0.0443 (17)	0.0497 (17)	−0.0040 (15)	−0.0004 (15)	0.0035 (15)
C3	0.071 (2)	0.0453 (19)	0.0460 (17)	0.0060 (18)	−0.0063 (16)	0.0010 (16)
C4	0.057 (2)	0.058 (2)	0.0406 (15)	0.0203 (18)	−0.0105 (15)	−0.0035 (15)
C5	0.0386 (16)	0.0567 (19)	0.0408 (16)	0.0048 (14)	0.0013 (13)	−0.0036 (14)
C6	0.0356 (14)	0.0472 (17)	0.0356 (14)	−0.0013 (12)	−0.0022 (11)	−0.0066 (12)
C1'	0.0315 (13)	0.0438 (16)	0.0375 (14)	−0.0017 (12)	−0.0029 (12)	−0.0064 (13)
C2'	0.0328 (15)	0.0548 (19)	0.0388 (15)	−0.0060 (13)	−0.0019 (12)	−0.0054 (14)
C3'A	0.078 (2)	0.053 (2)	0.0477 (17)	−0.0177 (19)	0.0093 (16)	−0.0044 (16)
C4'A	0.077 (4)	0.058 (3)	0.053 (2)	−0.004 (3)	0.016 (2)	0.002 (2)
C5'A	0.197 (8)	0.061 (3)	0.097 (4)	−0.029 (4)	0.070 (5)	0.002 (3)
C7	0.152 (5)	0.067 (3)	0.059 (2)	0.053 (3)	−0.006 (3)	0.0042 (19)

### Geometric parameters (Å, º)

**Table:**

O1—C2	1.351 (3)	C2'—C3'A	1.499 (5)
O1—C6	1.458 (3)	C2'—H2'	0.98 (3)
O2—C2	1.222 (4)	C3'A—C4'A	1.536 (5)
O4—C4	1.343 (4)	C3'A—H3'A	0.9900
O4—C7	1.451 (4)	C3'A—H3'B	0.9900
O1'—C1'	1.426 (3)	C4'A—C5'A	1.477 (7)
O1'—H1'O	0.85 (3)	C4'A—H4'A	0.9900
O2'—C2'	1.442 (3)	C4'A—H4'B	0.9900
O2'—H2'O	0.80 (4)	C5'A—H5'A	0.9800
C2—C3	1.447 (5)	C5'A—H5'B	0.9800
C3—C4	1.330 (5)	C5'A—H5'C	0.9800
C3—H3	0.98 (4)	C4'B—C5'B	1.57 (4)
C4—C5	1.486 (5)	C4'B—H4'C	0.9900
C5—C6	1.513 (4)	C4'B—H4'D	0.9900
C5—H5A	0.99 (3)	C5'B—H5'D	0.9800
C5—H5B	0.97 (4)	C5'B—H5'E	0.9800
C6—C1'	1.504 (4)	C5'B—H5'F	0.9800
C6—H6	0.98 (3)	C7—H7A	0.9800
C1'—C2'	1.524 (4)	C7—H7B	0.9800
C1'—H1'	0.94 (3)	C7—H7C	0.9800
			
C2—O1—C6	118.7 (2)	C3'A—C2'—H2'	105.9 (18)
C4—O4—C7	116.9 (3)	C1'—C2'—H2'	108.6 (17)
C1'—O1'—H1'O	102 (3)	C2'—C3'A—C4'A	112.2 (3)
C2'—O2'—H2'O	103 (3)	C2'—C3'A—H3'A	109.2
O2—C2—O1	116.3 (3)	C4'A—C3'A—H3'A	109.2
O2—C2—C3	124.5 (3)	C2'—C3'A—H3'B	109.2
O1—C2—C3	119.2 (3)	C4'A—C3'A—H3'B	109.2
C4—C3—C2	119.7 (3)	H3'A—C3'A—H3'B	107.9
C4—C3—H3	126 (2)	C5'A—C4'A—C3'A	112.3 (4)
C2—C3—H3	113 (2)	C5'A—C4'A—H4'A	109.1
C3—C4—O4	126.4 (3)	C3'A—C4'A—H4'A	109.1
C3—C4—C5	121.1 (3)	C5'A—C4'A—H4'B	109.1
O4—C4—C5	112.5 (3)	C3'A—C4'A—H4'B	109.1
C4—C5—C6	109.7 (3)	H4'A—C4'A—H4'B	107.9
C4—C5—H5A	108.2 (17)	C4'A—C5'A—H5'A	109.5
C6—C5—H5A	108.9 (19)	C4'A—C5'A—H5'B	109.5
C4—C5—H5B	110.5 (19)	H5'A—C5'A—H5'B	109.5
C6—C5—H5B	109 (2)	C4'A—C5'A—H5'C	109.5
H5A—C5—H5B	110 (3)	H5'A—C5'A—H5'C	109.5
O1—C6—C1'	105.3 (2)	H5'B—C5'A—H5'C	109.5
O1—C6—C5	110.2 (2)	C5'B—C4'B—H4'C	112.8
C1'—C6—C5	115.3 (2)	C5'B—C4'B—H4'D	112.8
O1—C6—H6	106.3 (15)	H4'C—C4'B—H4'D	110.3
C1'—C6—H6	110.7 (15)	C4'B—C5'B—H5'D	109.5
C5—C6—H6	108.7 (15)	C4'B—C5'B—H5'E	109.5
O1'—C1'—C6	106.9 (2)	H5'D—C5'B—H5'E	109.5
O1'—C1'—C2'	111.5 (2)	C4'B—C5'B—H5'F	109.5
C6—C1'—C2'	113.4 (2)	H5'D—C5'B—H5'F	109.5
O1'—C1'—H1'	112.6 (16)	H5'E—C5'B—H5'F	109.5
C6—C1'—H1'	106.5 (15)	O4—C7—H7A	109.5
C2'—C1'—H1'	106.0 (16)	O4—C7—H7B	109.5
O2'—C2'—C3'A	110.9 (3)	H7A—C7—H7B	109.5
O2'—C2'—C1'	109.1 (2)	O4—C7—H7C	109.5
C3'A—C2'—C1'	113.1 (3)	H7A—C7—H7C	109.5
O2'—C2'—H2'	109.1 (18)	H7B—C7—H7C	109.5
			
C6—O1—C2—O2	170.8 (3)	C4—C5—C6—C1'	−169.6 (2)
C6—O1—C2—C3	−11.9 (4)	O1—C6—C1'—O1'	64.9 (3)
O2—C2—C3—C4	162.4 (3)	C5—C6—C1'—O1'	−173.5 (2)
O1—C2—C3—C4	−14.7 (5)	O1—C6—C1'—C2'	−171.8 (2)
C2—C3—C4—O4	−174.4 (3)	C5—C6—C1'—C2'	−50.2 (3)
C2—C3—C4—C5	4.7 (5)	O1'—C1'—C2'—O2'	68.4 (3)
C7—O4—C4—C3	0.3 (5)	C6—C1'—C2'—O2'	−52.4 (3)
C7—O4—C4—C5	−178.8 (3)	O1'—C1'—C2'—C3'A	−55.5 (3)
C3—C4—C5—C6	28.2 (4)	C6—C1'—C2'—C3'A	−176.2 (3)
O4—C4—C5—C6	−152.6 (3)	O2'—C2'—C3'A—C4'A	63.0 (4)
C2—O1—C6—C1'	169.7 (2)	C1'—C2'—C3'A—C4'A	−174.2 (3)
C2—O1—C6—C5	44.8 (3)	C2'—C3'A—C4'A—C5'A	175.7 (5)
C4—C5—C6—O1	−50.7 (3)		

### Hydrogen-bond geometry (Å, º)

**Table:**

*D*—H···*A*	*D*—H	H···*A*	*D*···*A*	*D*—H···*A*
O1′—H1′*O*···O2^i^	0.85 (3)	1.93 (3)	2.778 (3)	177 (3)
O2′—H2′*O*···O2′^ii^	0.80 (4)	2.05 (4)	2.8178 (18)	163 (4)

Symmetry codes: (i) −*x*+1, *y*−1/2, −*z*+3/2; (ii) *x*−1/2, −*y*+1/2, −*z*+2.
